# Whole-Brain Network Models: From Physics to Bedside

**DOI:** 10.3389/fncom.2022.866517

**Published:** 2022-05-26

**Authors:** Anagh Pathak, Dipanjan Roy, Arpan Banerjee

**Affiliations:** ^1^National Brain Research Centre, Gurgaon, India; ^2^Centre for Brain Science and Applications, School of Artificial Intelligence and Data Science, Indian Institute of Technology, Jodhpur, India

**Keywords:** whole brain model, neural mass, neural field, network, neuroimaging, DTI, connectome

## Abstract

Computational neuroscience has come a long way from its humble origins in the pioneering work of Hodgkin and Huxley. Contemporary computational models of the brain span multiple spatiotemporal scales, from single neuronal compartments to models of social cognition. Each spatial scale comes with its own unique set of promises and challenges. Here, we review models of large-scale neural communication facilitated by white matter tracts, also known as whole-brain models (WBMs). Whole-brain approaches employ inputs from neuroimaging data and insights from graph theory and non-linear systems theory to model brain-wide dynamics. Over the years, WBM models have shown promise in providing predictive insights into various facets of neuropathologies such as Alzheimer's disease, Schizophrenia, Epilepsy, Traumatic brain injury, while also offering mechanistic insights into large-scale cortical communication. First, we briefly trace the history of WBMs, leading up to the state-of-the-art. We discuss various methodological considerations for implementing a whole-brain modeling pipeline, such as choice of node dynamics, model fitting and appropriate parcellations. We then demonstrate the applicability of WBMs toward understanding various neuropathologies. We conclude by discussing ways of augmenting the biological and clinical validity of whole-brain models.

## Physical Models of the Brain

Billions of years of evolution have invested the nervous system with tremendous complexity. Modern neuroscience has sought to understand this complexity as a hierarchical ladder that spans multiple spatial and temporal scales, starting from the interaction of biomolecules through to more complex structures like neurons and neural networks. Building on the pioneering work of Hodgkin and Huxley, significant progress took place toward the elucidation of the function of the single neuron. However, in spite of all the remarkable achievements at the single neuron level, relatively little is known about how populations of neurons coordinate with one another to facilitate cognitive processes. While it is fair to characterize neurons, or even individual dendrites as the canonical units of computation in the brain, it is evident that complex cognitive processes rely on interactions between several neural ensembles (McIntosh, 2004; Bressler and Tognoli, 2006)^1^ that are distributed across the cortex (Deco et al., 2008). Gaining an understanding of neural circuitry assumes vital importance not only for explaining cognition, but also for the treatment of various neurological diseases.

**Figure 1:**
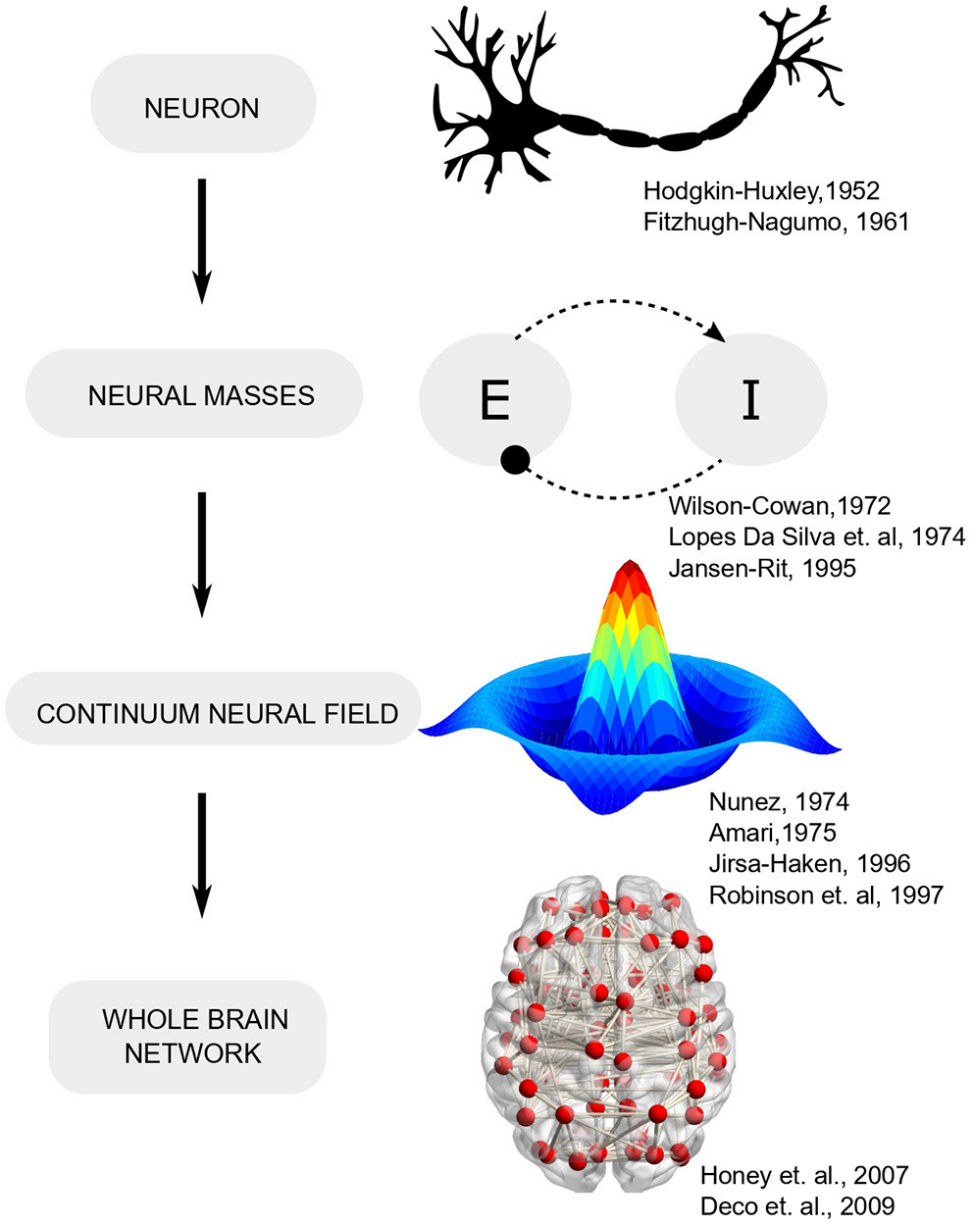
Evolution of computational neuroscience models from single neurons to network models.

Early efforts toward bridging the gap between single-neuron activity and circuit operation led to the formulation of neural mass models (Beurle, 1956; Wilson and Cowan, 1972) which conceptualized cortical activity as arising from the dynamic interplay of multiple neural populations (or masses) with excitatory-inhibitory feedback (Figure 1). Such models leverage the fact that while the spiking of individual neurons is highly irregular (even chaotic), the mean activity of neural ensembles obeys fairly low-dimensional dynamics (Deco et al., 2008) ^2^. Furthermore, mean-field descriptions of cortical tissue may be extended in space and endowed with spatial gradients in neural parameters that follow mathematically defined connectivity (Amari, 1977). Field theory, with its deep roots in physics, provides analytically tractable solutions and has been employed extensively in neuroscience to explain wide-ranging phenomena. A classic field-theoretic model is the one proposed by Amari, which considered lateral-inhibition to explain oscillatory waves and input-evoked transients (Amari, 1977). Two-dimensional field models support diverse phenomena such as spiral and target waves that are organized into complex checkerboard patterns, reminiscent of neural activity observed during different brain states (Ermentrout and Cowan, 1979; Jirsa and Haken, 1996).

By the turn of the century, neuroimaging modalities like PET and fMRI were being increasingly used to study cognition. The abstract nature of existing large-scale models made it difficult to exploit the rich datasets which were being churned in such experiments (Tagamets and Horwitz, 1998; Horwitz et al., 1999, 2000). Additionally, despite their success in providing theoretical accounts for neural phenomena such as traveling waves (Amari, 1977) or resting-state dynamics, Robinson et al. (2021) continuum field models had limited applicability in the clinical setting since crucial medical observables such as anatomical connectivity, functional correlations between brain areas or distribution of various cell types cannot be expressed in terms of mathematical expressions which can then be analytically solved within the field-theoretic framework. Therefore, it was deemed desirable to setup the neurodynamic model so that patient-specific neuroimaging data (e.g., DTI, fMRI connectivity) could be fused with simulations in order to facilitate precision medicine (Ritter et al., 2013; Deco and Kringelbach, 2014).

**Figure 2:**
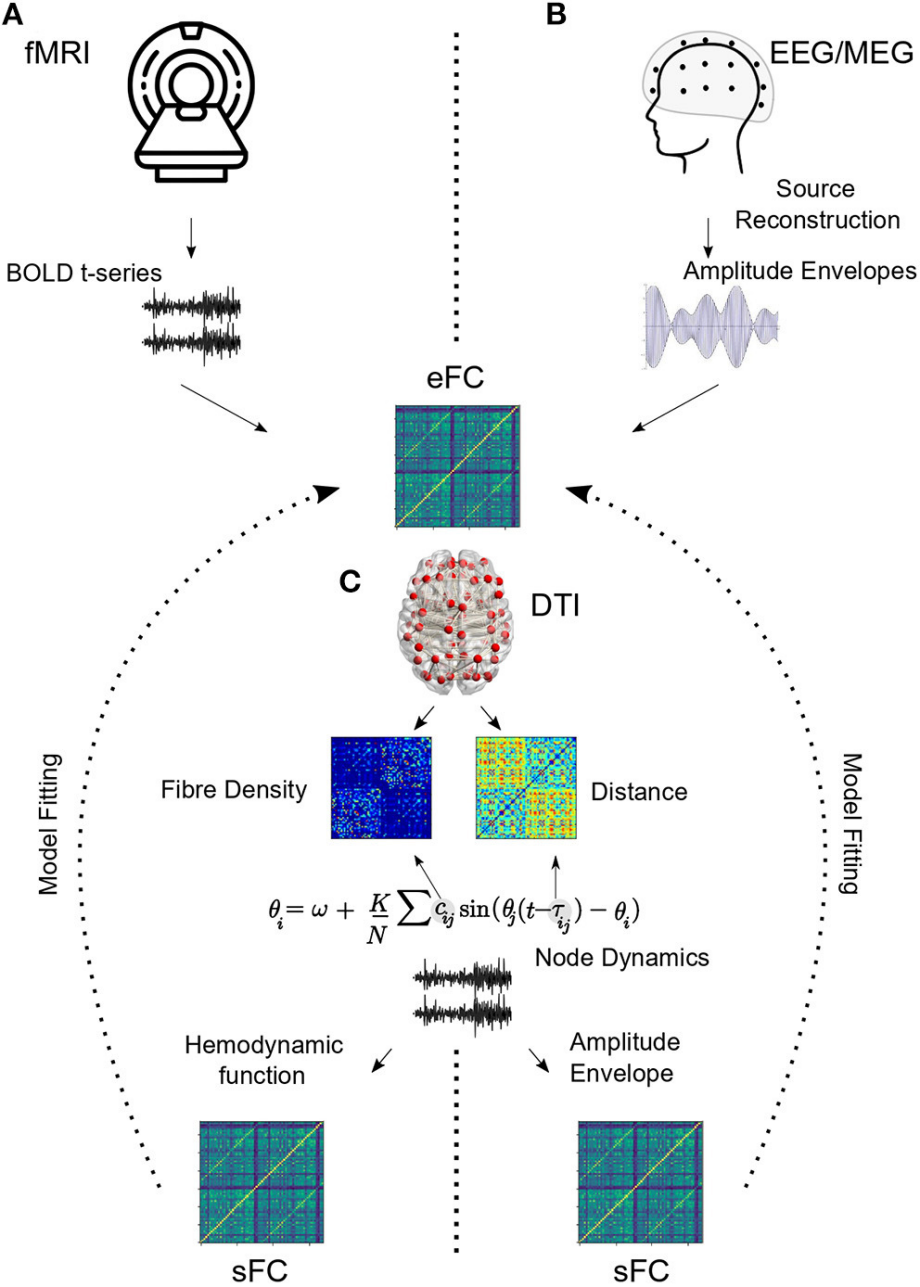
Model pipeline. **(A)** Inter-regional functional correlations (empirical functional connectivity or eFC) are extracted from resting state BOLD time-series. **(B)** For EEG/MEG, raw electrode/sensor time series are used to estimate source level activity, which is then bandpass filtered and hilbert transformed to obtain amplitude envelopes. Band specific functional connectivity is estimated from amplitude envelopes. DTI connectivity provides information about inter-regional white matter fiber density. Euclidean distances may be scaled to obtain temporal delay information. **(C)** Kuramoto model is used here to demonstrate how DTI information is incorporated into the node dynamics. For fMRI, an additional hemodynamic convolution is performed (Balloon-Windkessel) to project model output to BOLD time scales before estimating simulated FC (sFC). For EEG/MEG, amplitude envelopes are extracted and simulated FC is estimated. Model fitting procedures estimate model parameters based on eFC and sFC.

Within this framework, anatomical connectivity derived from diffusion MRI is used as a structural scaffold to simulate mesoscopic neural interactions (Horwitz et al., 2000; Honey et al., 2009). Nodes, representing mean-field activity of individual brain areas, evolve according to differential equations under the influence of coupling from other brain regions, external input and noise (Deco et al., 2009). Parameters representing biological or phenomenological properties of the nodes and edges are systematically varied, and time-series obtained for each run. For fMRI data, a further hemodynamic convolution is applied to the time-series and functional correlations (FC) are estimated from the resulting data (Deco and Kringelbach, 2014). Alternatively, for EEG/MEG studies, FC is estimated from amplitude envelopes that are extracted for each frequency band of interest and downsampled to correspond with BOLD time-scales (Hipp et al., 2012). Model fitting techniques are then utilized to obtain working points and regimes that best capture the corresponding empirical data. After model fitting, the researcher can ask how this system responds to various perturbations like external inputs (e.g., stimuli), noise or structural insults (e.g., lesions) (Figure 2).

Whole-brain models provide actionable insights into various neurological deficits (e.g., identifying optimal resection zone in epilepsy), while also retaining a link to fundamental dynamical and graph theoretic concepts like attractors, metastability, stochastic dynamics, chaos and modularity (Popovych et al., 2019). In the following we outline the major variables that need to be considered before establishing a successful WBM pipeline.

## Modeling Considerations

Whole-brain modeling has tremendous clinical applicability as it provides prognostic tools and predictive insights for a host of neurological diseases (Deco and Kringelbach, 2014). However, since not all brain pathologies have the same origin or mechanism, the models seeking to understand them are also customized according to the specific etiology of the disease (Table 1). Following E.P Box's adage- “all models are wrong, but some are useful,” system equations are set up keeping in mind the specific properties of the underlying clinical context at the expense of biological realism. For example, it may be unnecessary to include conduction delays in models seeking to fit fMRI data due to the widely differing time-scales between BOLD activity (seconds) and axonal propagation (milliseconds). On the other hand, delays assume vital importance when the object of study involves electrophysiological spectral coherence between neural oscillators. Broadly, establishing an effective whole- brain modeling pipeline essentially comes down to the following choices- parcellation scheme, node dynamics, model fitting technique and type of perturbation applied.

**Table 1:** List of studies employing WBMs to understand neuropathologies.

**References**	**Clinical context**	**Node dynamics**	**Model fitted to**	**Parcellation(N)**	**FC (dynamic or static)**
Alstott et al. (2009)	Lesion	Neural mass model	BOLD FC	Hagmann (998)	Static
Demirtaş et al. (2017)	AD	Hopf Normal Form (Stuart-Landau)	BOLD FC	78 Cortical	Static
Vattikonda et al. (2016)	Stroke	Dynamic Mean Field (DMF)	BOLD FC	Desikan Killainy (68), Hagmann (998)	Static
Jirsa et al. (2017)	Epilepsy	Epileptor	SEEG spectral power	EZ/PZ	Static
Nakagawa et al. (2014)	Aging	Dynamic Mean Field (DMF)	BOLD FC	Modified CoCoMac (74)	Static
Deco et al. (2017)	Psychadelics	Dynamic Mean Field (DMF)	BOLD FC	Automatic Anatomical Labelling (90)	Dynamic
Griffiths et al. (2020)	Stimulation	Thalamocortical Motif	AEC MEG	Lausanne Scale 1 (68)	Static
López-González et al. (2021)	Disorders of Consciousness (DOC)	Hopf Normal Form (Stuart-Landau)	BOLD phase synchrony	Shen (214)	Dynamic
Tait et al. (2021a)	Seizure Propensity in AD	Theta Model	EEG phase locked FC	Brainnetome (40)	Static
Hellyer et al. (2015)	Traumatic Brain Injury	Kuramoto Oscillator	BOLD FC	Desikan-Killainy (68)	Static
Cabral et al. (2013)	Schizophrenia	Linear relaxation process	BOLD FC	AAL (90), Hagmann (66)	Static
Yang et al. (2014)	Schizophrenia	Dynamic Mean Field (DMF)	BOLD FC	Hagmann (66)	Static

### Structural Connectivity Matrices and the Role for Parcellation

Firstly, thousands of voxels are reduced to only a few relevant areas of interest. Diffusion imaging is performed to extract anatomical connectivity matrices (Box 1). Connectivity matrices specify fiber density across various white matter tracts. A crucial decision at this stage is the choice of a parcellation scheme for obtaining an adjacency matrix. In the absence of a general consensus on what constitutes a “good” parcellation, one must consider carefully how the parcellation scheme may affect the WBM pipeline. Parcellation dictates the spatial resolution and topology of the model. Topological properties are known to be affected by the spatial scale of the parcellation used (Zalesky et al., 2010). Zalesky et al. (2010) demonstrate that while the basic properties of network topology such as scale-freeness or small-worldness remain invariant across spatial scales, the extent of these properties significantly varies between parcellations. Modeling has further corroborated that significant variability exists in graph-theoretical attributes of network dynamics as a function of the parcellation scheme (Domhof et al., 2021). Significant inter-parcellation variability also exists in resting-state dynamical models (Fornito et al., 2010). Additionally, since the computation time for whole-brain models scales with the number of coupled differential equations (same as the nodes in the network), finer-grained parcellations may be computationally cumbersome to solve. Further, diffusion MRI (dMRI) techniques are biased against short-range and intracortical connectivity, which may have significant ramifications for simulated dynamics (Proix et al., 2016). Highly granular parcellation schema may also lead to redundancy and rank-deficiency during source reconstruction (Tait et al., 2021b).

Box 1 **Estimating anatomical connectivity**

*In-vivo* estimation of white matter structural connectivity is enabled by diffusion magnetic resonance imaging (dMRI). Broadly, dMRI approaches measure the preferential direction of diffusion of water molecules in brain tissue. Computational algorithms estimate fiber orientations (streamlines) from dMRI data using a process known as tractography. Streamlines are counted and averaged according to pre-defined brain parcellations to yield adjacency graphs, which can then be submitted as input for whole-brain models. Computational libraries that perform tractography include FSL (Jenkinson et al., 2012), MRtrix (Tournier et al., 2012), BrainSUITE (Shattuck and Leahy, 2002), and DSI studio (Yeh et al., 2013). Considerable variability may exist in the output of different libraries due to differences in the choice of diffusion models, model parameters and tractographic algorithms. Thus, optimal algorithm selection remains an active area of research (Bastiani et al., 2012; Zhan et al., 2015; Petrov et al., 2017).

Broadly, atlases bin brain areas on the basis of either anatomical or functional similarities. Commonly used anatomical criteria for parcellating brain regions include gross anatomy, cytoarchitecture, myeloarchitecture, chemoarchitecture and gene expression profiles (Nowinski, 2021). By contrast, functional atlases utilize resting state or task-related functional correlations to allocate ROIs (Craddock et al., 2012; James et al., 2016). Anatomical atlases are known to fare poorly in comparison to functional atlases when it comes to reproducing FC patterns at the voxel scale (Craddock et al., 2012). Since functional homogeneity is a crucial precondition for modeling ROI dynamics, this would argue for the superiority of functional over anatomical parcellations for whole-brain modeling (Craddock et al., 2012). On the other hand, with anatomically defined ROIs it is easier to interpret results in the light of extant neuroscience literature. Therefore, multimodal atlases which integrate anatomical and functional criteria may offer a suitable tradeoff to ensure functional homogeneity while retaining anatomical specificity in whole-brain analysis (Glasser et al., 2016).

Ultimately, the scope of the study dictates the choice of parcellation. For example, it may be crucial to include sub-cortical nodes where the primary pathology may involve subcortical structures like the thalamus (Ji et al., 2016; Bazin et al., 2020).

### Node Dynamics

**Figure 3:**
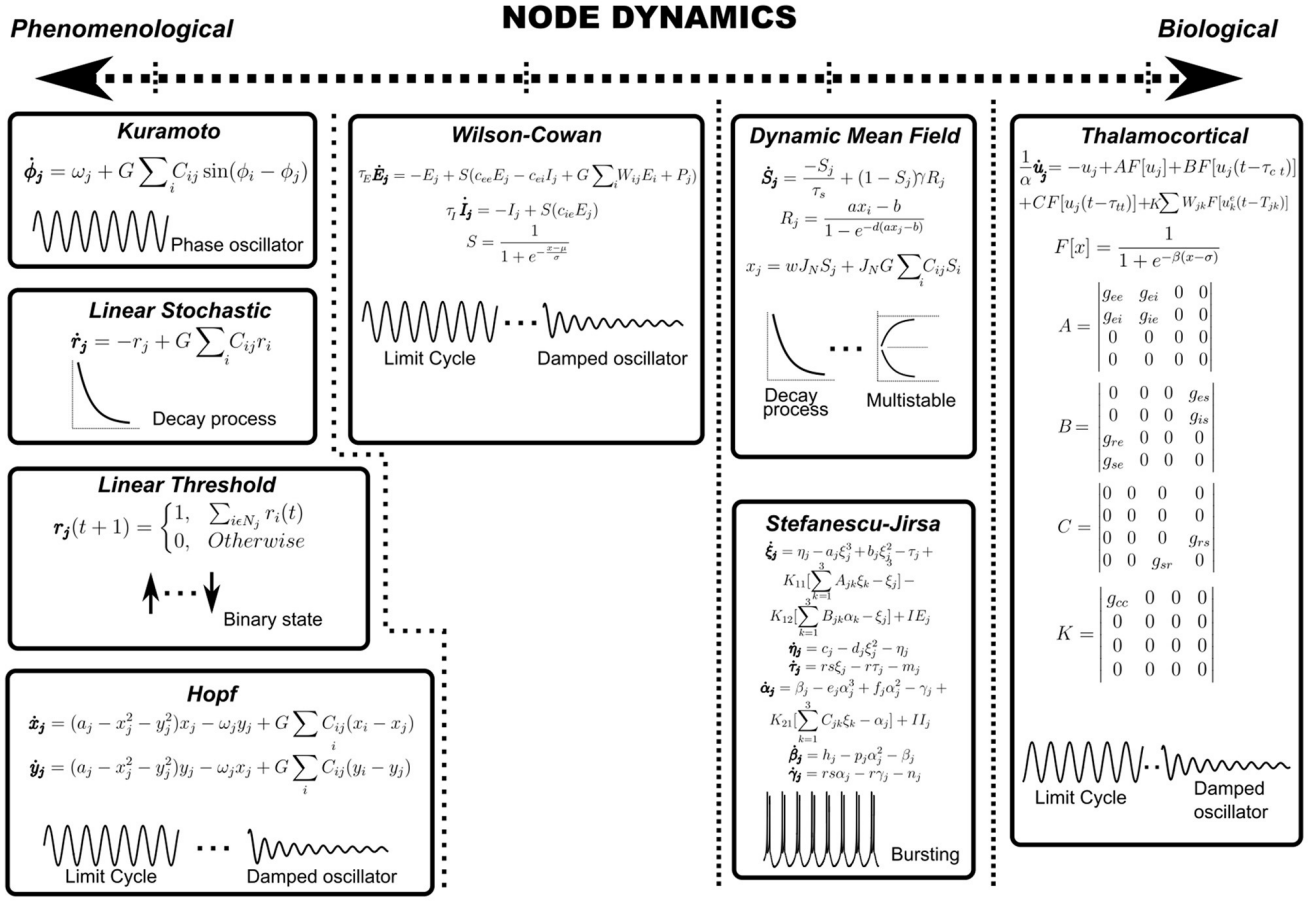
Node dynamics. Node dynamics may be categorized on the axis of biological validity. Purely phenomenological models such as Kuramoto, Linear Stochastic, Linear Threshold or the normal form of Hopf bifurcation may be regarded as toy models which seek to focus on a conceptual aspect of underlying phenomena at the expense of biological realism. On the other hand, it may be necessary to impart nodes with biologically detailed dynamics (SJ3D or Thalamocortical motifs) to explain a certain biological feature of the research problem. Further, nodes may be categorized on the type of underlying dynamics, such as damped oscillators, limit cycle oscillators, relaxation processes, multistable solutions or bursting patterns (represented schematically).

Node dynamics consist of differential equations specifying the temporal evolution of the population activity of each region of interest (ROI). Each anatomically defined node may potentially consist of thousands of neurons and therefore, the dynamics of the ensemble is reduced to a low-dimensional description using mean-field formalisms. For example, Deco et al. reduce a spiking neuron model with synaptic conductance to yield a dynamic mean-field model that is subsequently used to specify node dynamics (Deco et al., 2013; Roy et al., 2014). Examples of node dynamics may range from the simple phenomenological ones, such as the Kuramoto model (Breakspear et al., 2010) or the normal form of the supercritical Hopf bifurcation (Lord et al., 2017) to the more biologically inspired ones such as the Wilson-Cowan model (Wilson and Cowan, 1972) or thalamocortical motifs (Griffiths et al., 2020) (see Figure 3 and Table 1). For example, the Kuramoto model reduces node dynamics to a phase variable, which evolves according to a natural frequency and a sinusoidal interaction term (Breakspear et al., 2010). On the other hand, both asynchronous and synchronous dynamics can be captured in the same set of equations in bifurcation models that can possess a relaxation solution (damped oscillations) or limit cycle solution (self-sustained oscillations) depending on the value of the bifurcation parameter (Figure 3) (Lord et al., 2017). Most computational studies model average functional connectivity, however, brain dynamics is also marked by transitions in the patterns of functional connectivity with time. Switching between FC configurations (FC state) requires node dynamics to possess multistable solutions which may be imparted through the addition of non-linear terms in model equations (Deco et al., 2013; Hansen et al., 2015).

Heterogeneity in node dynamics may be introduced by assigning multiple oscillatory frequencies. For example, Deco et al. (2017) show that models utilizing multiple natural frequencies confirm better to empirical rsMEG networks. Similarly, Roberts et al. devise a principled approach for natural frequency allocation by scaling frequencies by the topological degree of each node (Gollo et al., 2017). For non-oscillatory node dynamics, temporal heterogeneity may be introduced by modulating exponential decay rates or synaptic time constants (Figure 3).

Another decision to be made at this step is the inclusion of transmission delays (Nakagawa et al., 2014). Computational models have demonstrated the value of including transmission delays, particularly in explaining oscillatory activity at electrophysiological time scales (Banerjee and Jirsa, 2007; Deco et al., 2009). Neural delays are known to play a crucial role in motor control, particularly in bimanual coordination (Banerjee and Jirsa, 2007) and in explaining perceptual variability in multi-sensory integration (Thakur et al., 2016). Conduction delays, on the order of a few milliseconds, can flip the phase relationship between two gamma oscillators from in-phase to out-of-phase (Pajevic et al., 2014). Network delays crucially dictate oscillation frequency (Niebur et al., 1991; Petkoski and Jirsa, 2019; Pathak et al., 2021) and propagation of cortical traveling waves (Ermentrout and Kleinfeld, 2001). Delays can even cause the complete cessation of self-sustained oscillations (amplitude death) in networks of coupled limit-cycle oscillators (Reddy et al., 1998). On the other hand, under certain conditions, time delays may also enhance neural synchrony (Dhamala et al., 2004).

Conduction delays may be estimated by scaling cortico-cortical tract lengths by conduction velocity, which is usually parametrically varied between 1-30 m/s, in accordance with experimental studies (Swadlow, 1982). However, given the millisecond scale of delays involved, it may be redundant to include delays in cases where the object of interest is fMRI BOLD time scales.

### Model Fitting

Typically, whole-brain models aim to explain data collected at fMRI BOLD or electrophysiological (EEG, MEG, sEEG) time scales. Functional time series collected from fMRI experiments are used to estimate inter-areal functional connectivity. For EEG or MEG, pre-processed signals are bandpass filtered in various frequency bands of interest and Hilbert-transformed to extract amplitude envelopes, which are then used to estimate functional connectivity (Hipp et al., 2012; Deco et al., 2017). For both fMRI and EEG/MEG, typically static correlations (presuming stationarity) are employed to estimate model fit. However, recent work has strongly argued that static measures fail to capture the rich, higher-order dynamics inherent in neuroimaging data, and therefore, have advocated the use of dynamic measures of functional connectivity (dFC) (Hutchison et al., 2013; Preti et al., 2017). dFC may be estimated through a windowed manner, or through techniques not requiring arbitrarily chosen temporal windows (Cabral et al., 2017). For dFC analysis, every time step (or time window) has a characteristic FC pattern associated with it. One way to perform model fitting for dFC is by collapsing this 3D data structure (ROI*ROI*time) to a 2D matrix (time*time) consisting of correlations between the leading eigenvectors at each time point or window; the resulting matrix may be considered as the object of model fitting (Cabral et al., 2017).

Model parameters are systematically varied and simulated FCs (static or dynamic) are estimated for each parametric set. Estimation of the optimal parameter set (often referred to as the dynamic working point of the system), offering closest concordance with empirical FC may be achieved by minimizing an error function or by maximizing correlation between empirical and simulated FCs (Deco and Kringelbach, 2014). Bayesian modeling is often employed to estimate parameters associated with the underlying generative models (Vattikonda et al., 2016; Hashemi et al., 2020). Here, models are initialized with a randomly chosen parameter set; stochastic gradient descent is then used to update model parameters.

Instead of FC, one could alternatively perform model fitting against other empirical features of the data. For example, Jirsa et al. (2017) develop a personalized epileptic brain model by estimating model parameters from the spectral distribution of stereotactic (SEEG) electrodes. Indeed, it is even possible to do away with model fitting altogether when the research question is of a qualitative nature. For example, Mejias and Wang (2022) simulate a large-scale model of primate neo-cortex to elucidate the emergence of distributed attractor states subserving various internal processes. In such studies, explaining the salient aspects of the underlying system takes precedence over precise model fitting (Mišić et al., 2015; Mejias and Wang, 2022).

### Perturbation

After successfully fitting the model to relevant empirical data, it is desired to introduce various perturbations to the model in order to understand the fallout of various pathological scenarios (Deco et al., 2015). For example, in the case of stroke or TBI, one would like to induce partial or complete lesions at various network nodes and study the differential contribution of node topology in disease progression (Alstott et al., 2009; Vattikonda et al., 2016). Since the thrust here is to understand recoverability, individual node dynamics can be endowed with plasticity mechanisms that homeostatically regulate firing rates (Vattikonda et al., 2016; Abeysuriya et al., 2018; Páscoa Dos Santos and Verschure, 2021). Similarly, stimulation protocols require providing current input to specific nodes in the network to study network response (Griffiths et al., 2020). Epilepsy models require altering node dynamics such as channel properties or neurotransmitter concentrations to model seizure spread from the seizure onset zone (SOZ) (Jirsa et al., 2017). Levels of consciousness in whole-brain models can be manipulated by adjusting neural gain, say, mediated by subcortical structures (Shine, 2021).

## Clinical Applications

### Modeling Seizure Propagation

Epilepsy is marked by the occurrence of frequent seizures, which often spread from an onset zone to other distal areas along white matter tracts. In some cases, this necessitates the surgical resection of epileptogenic tissue. Due to the obvious role of network dynamics and structural topology, epilepsy is particularly well-suited for whole-brain modeling, as described in previous sections (Engel Jr et al., 2013; Taylor et al., 2014; Jirsa et al., 2017). Since surgery carries obvious risks, it is desirable to minimize the extent of resected tissue. Jirsa et al. show that personalized whole-brain modeling can be used to aid medical decision making for optimal surgery (Vattikonda et al., 2016; Jirsa et al., 2017). Patient-specific brain connectivity is integrated to model empirical EEG data for the identification of the epileptogenic zone (EZ). This technique is particularly useful for instances where conventional methods for EZ identification provide sub-optimal results due to a lack of a clear MRI lesion (Hashemi et al., 2020). Recently, whole-brain modeling has also been used to explain seizures in non-epileptic conditions as well. For example, it is known that patients with Alzheimer's disease are about 6-10 times more likely to develop seizures as compared to the normal population (Pandis and Scarmeas, 2012). Tait et al. (2021a) using a whole-brain pipeline, find that functional connectomes of AD patients show a greater propensity to transition into seizure states as compared to healthy connectomes. Here individual nodes in the network are modeled as phase oscillators capable of producing neuronal spiking in response to inputs. By systematically varying the excitability parameter of individual nodes, the authors show that AD connectomes are more ictogenic as compared to control connectomes for a wide range of excitatory input (Tait et al., 2021a).

### Lesions

**Figure 4:**
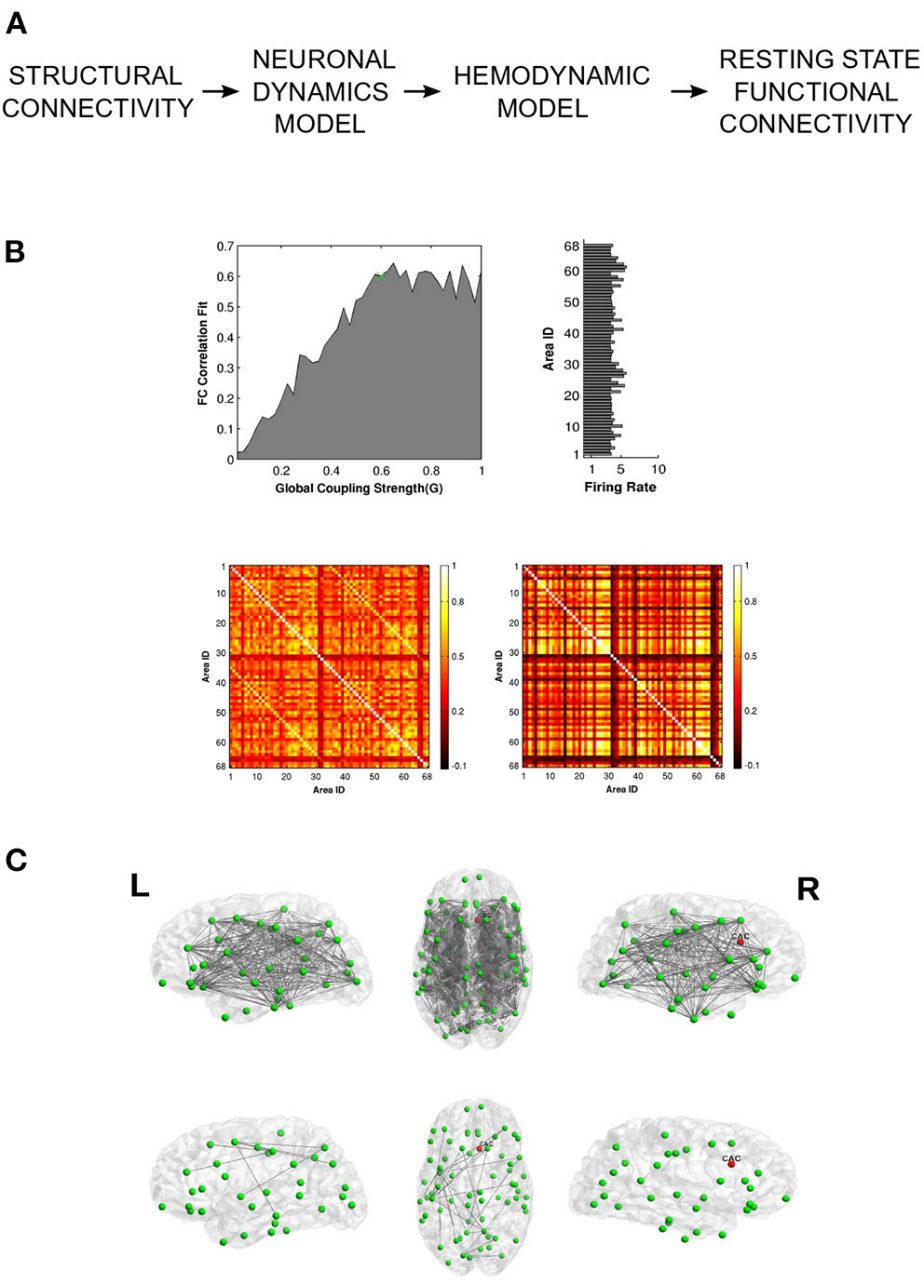
Vattikonda and colleagues use a whole-brain pipeline to elucidate network recovery after lesional insult. **(A)** DTI structural connectivity is used in conjunction with mean field node dynamics (with feedback inhibition) to generate ROI time series which are then convolved with hemodynamic function to obtain simulated BOLD time-series. Resting state FC is calculated using pearson correlation. **(B)** Global working point of the system is estimated by comparing simulated FC with empirical FC. Histogram shows the ROI-wise average firing rate for optimal global coupling. Heat Maps show empirical and optimal simulated FC (averaged across subjects). **(C)** Network recovery takes place due to local plasticity implemented through feedback inhibition. Here lesion was introduced in the right caudal anterior cingulate node. The top 3 figures display connections that have significantly changed before re-establishing local E-I balance. The bottom 3 figures represent the connections that have significantly changed after re-establishing local E-I balance. Adapted from Vattikonda et al. (2016), with permission from the authors.

Neural tissue undergoes lesioning due to various factors like traumatic brain injury, stroke or neurodegenerative diseases (Alstott et al., 2009). Focal lesions can cause disruptions in large-scale functional connectivity, leading to severe cognitive and behavioral impairment. Alstott et al. (2009) demonstrate that the extent and severity of functional deterioration depends on the topological profile of the lesioned nodes, with nodes occupying the most central position causing the greatest network deficit upon lesioning. Vattikonda et al. (2016) extend this idea to gauge potential recoverability from stroke induced lesions by endowing node dynamics with an inhibitory plasticity mechanism that can rescue neural firing rates in response to structural insult (Figure 4). Recently, Good et al. used whole-brain modeling to predict the chronic outcomes following traumatic brain injury. Their approach, which utilizes the Virtual Brain simulation platform (Sanz Leon et al., 2013), is able to distinguish semiacute mild to moderate TBI patients from a control group (Good et al., 2022). The effect of lesions on segregative and integrative tendencies can be quantified using WBMs. For example, Hellyer et al. (2015) estimate metastability- a measure of segregation and integration, and find disrupted metastable dynamics in patients with traumatic brain injury (TBI). By simulating a network of phase oscillators on topology specified by connectomes obtained from TBI patients, the authors demonstrate how structural disconnection can lead to a reduction in metastable brain dynamics. These observations provide a mechanistic explanation for the significant reductions in cognitive flexibility and information processing, often seen in patients recovering from TBI lesions. Váša et al. (2015) highlight the usefulness of computational lesion studies by demonstrating how graph theoretic properties of network nodes such as modularity determine synchrony and metastability in response to virtual lesioning. The authors find that lesions to nodes with high eigenvector centrality or to nodes which connect segregated modules lead to a decrease in global synchrony along with an increase in global metastability (Váša et al., 2015).

### Alzheimer's Disease

Alzheimer's Disease (AD) has traditionally been regarded as a disease of the gray matter, however, recent neuroimaging studies have implicated white matter abnormalities in the pathogenesis of AD (Sachdev et al., 2013). This is reflected in aberrant functional connectivity patterns observed in preclinical populations. Demirtaş et al. (2017) fit a whole-brain model to healthy controls; the model parameters thus obtained are then systematically varied to generate FCs which match empirical FCs seen in preclinical AD, Mild Cognitive Impairment and AD. The authors find that simulated FCs mimic pathological FCs as the individual node dynamics is shifted toward damped oscillations by altering the bifurcation parameter (Demirtaş et al., 2017). Stefanovski et al. (2019) fuse PET-derived Amyloid beta levels with averaged healthy connectomes to shed light on possible pathogenetic mechanims of AD. In this model, Amyloid beta levels modulate the regional Excitation/Inhibition balance, providing a mechanistic explanation for EEG alterations in AD. Further, their whole-brain approach provides therapeutic insights by accounting for large-scale functional reversibility of EEG alterations by modeling the effect of memantine (NMDA receptor antagonist) on local neural populations (Stefanovski et al., 2019). Recently, whole-brain network models have also been utilized for virtual data completion to augment multimodal AD datasets such as the Alzheimer's Disease Neuroimaging Initiative (ADNI) dataset (Arbabyazd et al., 2021).

### Schizophrenia

The dysconnection hypothesis posits that symptoms of Schizophrenia are best characterized as emerging from functional, rather than anatomical disconnection (Friston, 1998). In line with this assertion, several studies have observed extensive decrease in resting state functional connectivity of patients, pointing to disrupted integration between segregated brain areas (Lynall et al., 2010). Cabral et al. (2013) employ structural connectivity matrices obtained from adolescent patients with early onset schizophrenia and show that functional disruptions associated with Schizophrenia are better explained by reductions in global coupling rather than structural differences, in line with the dysconnection hypothesis. Yang et al. (2014) use whole-brain modeling to show that widely reported differences in global brain signal (GBS) in resting state fMRI of patients may be explained by changes in the net strength of overall brain connectivity in schizophrenia, further corroborating dysconnection. Anticevic et al. (2012) use whole-brain models to identify the role for glutamate in establishing large-scale functional patterns associated with Schizophrenia. Their whole-brain approach, which allows for the introduction of pharmacological manipulations, provides a framework for understanding the role of NMDA-mediated disruption of cortical excitation/inhibition balance and its role in producing the cognitive symptoms of schizophrenia.

### Disorders of Consciousness

Loss of consciousness is either temporary, like in deep sleep or anesthesia- or permanent- like in brain injury or other Disorders of Consciousness (DoC). Efficient classification of brain states as either reversibly or irreversibly unconscious is needed to advance therapeutics. One way to gauge whether a certain unconscious state is transient or permanent is from the response of that state to externally provided perturbation. Recently, whole-brain models have been used to characterize brain states in terms of their stability toward perturbation. Sanz Perl et al. (2021) demonstrate that perturbational analysis can complement machine-learning based algorithms which classify different states of consciousness. López-González et al. (2021) use structural connectivity from healthy and injured subjects to show that low-level states of consciousness are associated with decreased network interactions, leading to segregation of synchronization patterns in fMRI brain dynamics. Segregative tendencies are found to be associated with the global coupling parameter that scales the weights of the SC matrix.

## Promises and Pitfalls

Since whole-brain approaches leverage neuroimaging modalities for modeling neural dynamics, future improvement is contingent upon parallel advances in diffusion imaging, functional imaging and signal processing techniques. Here, we discuss a few directions that can significantly augment current neurocomputational models.

One potential avenue for enriching current whole-brain models is by improving the estimation of structural adjacency matrices. For example, DTI derived structural matrices are bidirectional, whereas actual white matter fibers have a well-defined point of origin and termination which imparts directionality and has obvious consequences for the emerging dynamics. Additionally, current protocols for structural estimation rely on the number of streamline (NOS) methods which reconstruct structure by counting the number of streamlines between ROI pairs. Although showing concordance with tract-tracing, the NOS method has inherent limitations as it does not consider other biologically crucial parameters like conduction speeds. Thus, estimation of structural connectivity matrices can be further improved by inclusion of myeloarchitecture, since myelin plays a crucial role in determining conduction speeds across axons (Boshkovski et al., 2021). One way to achieve this is by weighting the connectome with longitudinal relaxation rate (R1), which is sensitive to myelin. Boshkovski et al. (2021) show how including myelin weighted structural connectomes is successful at separating transmodal regions from unimodal regions . Inclusion of myelin in network simulations has particular application at electrophysiological time-scales where phase lags often arise due to finite conduction delays (Petkoski and Jirsa, 2019). g-ratio, which quantifies the ratio between axon diameter and myelin thickness, has recently been shown to be estimable through MRI protocols (Berman et al., 2019; Drakesmith et al., 2019). *In vivo* g-ratio mapping has the potential to provide novel insights into cortical conduction speeds (Berman et al., 2019; Drakesmith et al., 2019). Another method being currently explored for the estimation of cortical conduction velocity uses direct electrical stimulation to measure the propagation of electrophysiological responses across the cortex in patients implanted with intracranial electrodes for seizure monitoring (David, 2021). Harnessing signal propagation information has far-reaching applications, especially toward understanding various demyelinating disorders such as multiple sclerosis.

Further augmentation of whole-brain connectomes comes from incorporating neuromodulatory information (Deco et al., 2018; Kringelbach et al., 2020; Naskar et al., 2021). Multi-modal integration between diffusion imaging (structural connectivity) and PET (receptor density) allows for the infusion of dynamic information to static network models. Kringelbach et al. (2020) have employed a similar pipeline to model the bidirectional interaction of neuronal and neurotransmitter systems that sheds light on the action of psilocybin on human resting state activity. Understanding large-scale functional impact of neuromodulation is of primary importance to computational neuropsychiatry given the therapeutic potential of psychedelics in the treatment of anxiety and depression (Deco et al., 2018).

Another limitation of most current large-scale models is the absence of sub-cortical nodes in the network. This is partly due to inadequate resolution offered by most atlases at the sub-cortical level. Additionally, various sub-cortical structures (e.g., thalamus) possess unique network architecture, requiring the development of specialized node dynamics (see thalamocortical motifs, Figure 3). Here we direct the interested reader to some recent efforts toward addressing this lacuna (see Shine et al., 2018; Griffiths et al., 2020; Shine, 2021). Future developments in high field strength imaging, sub-cortical node dynamics and parcellations offer the possibility of having truly whole-brain models.

Despite substantial progress in the field, most successful whole-brain models are limited to either BOLD (fMRI) or BOLD time-scale (amplitude envelopes) functional correlations. Lacunae exist about the extent to which whole-brain models may explain phenomena at electrophysiological time-scales, especially since neural oscillations are so well-linked to the underlying white matter structure and are crucial to cognition (Chu et al., 2015; Hindriks et al., 2015). Signal processing techniques that circumvent or correct for volume/field spread effects which tend to contaminate electrophysiological data would go a long way toward informing whole-brain modeling (Hipp et al., 2012).

Similarly, the present thrust of whole-brain approaches is oriented toward modeling recordings while participants are not engaged in overt cognition, aka resting-state (Biswal et al., 1995; Deco et al., 2011; Popovych et al., 2019). Going forward, whole-brain models could also be explored for explaining various tasks and learning paradigms, requiring richer node dynamics with neuromodulatory and plasticity properties (Abel et al., 2013; Maniglia and Seitz, 2018; Zhang et al., 2021). Finally, foundational discoveries in graph theory and non-equilibrium physics will continue to offer new insights into the mechanistic underpinnings of large-scale brain dynamics.

## Conclusion

Computational neuroscience aims to understand the biophysical principles underlying brain function. Many cognitive phenomena crucial for understanding the brain in health and disease evolve at the mesoscopic scale, where the firing patterns of individual neurons get averaged out, thereby offering an opportunity for radical dimensionality reduction. Whole-brain models leverage new advances in neuroimaging techniques to simulate white matter-mediated large-scale brain networks that underlie cognitive and behavioral processes in health and disease. In this article, we provided a brief outline of how coarsely grained models of brain dynamics may be employed to gain insights into the mechanistic underpinnings of brain dynamics, an endeavor central to the emerging field of computational psychiatry. We summarized the various choices at hand for the successful implementation of whole-brain pipelines and discussed those in the context of relevant case studies. Researchers must be mindful of how the choices of parcellation, node dynamics, model fitting procedure and perturbation impact the modeling pipeline and relate to the underlying scientific objective of the study.

We discussed how large-scale modeling has provided crucial insights into the biology of various neuropathologies like Epilepsy, Stroke, Traumatic Brain Injury, Alzheimer's Disease, Schizophrenia and Disorders of Consciousness. Like any emerging field, whole-brain modeling also requires further developments to tap into its full potential and we provided methodological and technical recommendations for the growth of large-scale modeling. Improvements in structural brain imaging and signal processing techniques can significantly enhance the accuracy of neurocomputational models. Similarly, the inclusion of sub-cortical, neurotransmitter and myelination information can lead the field toward truly whole-brain models. Going forward, the continued development of new computational platforms like the Virtual Brain simulator (Sanz Leon et al., 2013) is likely to bridge the gap between theory and implementation, making whole-brain modeling more accessible to medical professionals and biologists alike. In closing, whole-brain models are the newest addition to the rich arsenal of computational neuroscience techniques and promise to usher in a new era in personalized medicine.

## Author Contributions

AP and AB conceived the study. AP wrote the first draft. AP, DR, and AB wrote and revised manuscript. All authors contributed to the article and approved the submitted version.

## Funding

DR, Ramalingaswami Fellowship, Department of Biotechnology, Government of India, Award ID: BT/RLF/Re-entry/07/2014. DR, Department of Science and Technology (DST), Ministry of Science and Technology, Government of India, Award ID: SR/CSRI/21/2016. AB, Ministry of Youth Affairs and Sports, Government of India, Award ID: F.NO.K-15015/42/2018/SP-V. AB, NBRC Flagship program, Department of Biotechnology, Government of India, Award ID: BT/MED-III/NBRC/Flagship/Flagship2019.

## Conflict of Interest

The authors declare that the research was conducted in the absence of any commercial or financial relationships that could be construed as a potential conflict of interest.

## Publisher's Note

All claims expressed in this article are solely those of the authors and do not necessarily represent those of their affiliated organizations, or those of the publisher, the editors and the reviewers. Any product that may be evaluated in this article, or claim that may be made by its manufacturer, is not guaranteed or endorsed by the publisher.

## Footnotes

^1^Several thousand neurons exhibiting coordinated firing patterns.

^2^In mean field models, coarse-grained variables representing ensemble activity such as the population firing rate are used to track the evolution of the system.

## Abbreviations

MRI: Magnetic resonance imaging
WBM: Whole-Brain Model
ROI: Region of Interest
BOLD: Blood oxygen level dependent
EEG/MEG: Electro/Magneto encephalography
FC: Functional connectivity.
